# Uptake of Tele-Rehabilitation in Finland amongst Rehabilitation Professionals during the COVID-19 Pandemic

**DOI:** 10.3390/ijerph18084383

**Published:** 2021-04-20

**Authors:** Tuija Heiskanen, Hanna Rinne, Sari Miettinen, Anna-Liisa Salminen

**Affiliations:** Kela Research, Social Insurance Institution of Finland, 00250 Helsinki, Finland; hanna.rinne@kela.fi (H.R.); sari.miettinen@kela.fi (S.M.); anna-liisa.salminen@kela.fi (A.-L.S.)

**Keywords:** tele-rehabilitation, telepractice, COVID-19 pandemic, rehabilitation professionals, digital health, mRehab, physiotherapy, occupational therapy, psychotherapy, speech and language therapy

## Abstract

The COVID-19 pandemic has challenged rehabilitation professionals to provide therapy through telepractice. The aims of this study were to investigate and compare the uptake of tele-rehabilitation (TR) in Finland amongst different rehabilitation professions during the COVID-19 pandemic as well as potential differences between professions in carrying out TR. In addition, the goal was to explore in more depth therapists’ views about the features that work and challenges of TR. A total of 850 therapists in the physio-, occupational-, speech and language-, and psychotherapy professions participated in the survey that included both quantitative and open-ended questions. The results show that 52% of all the therapists who participated in this study did take up TR with all or most of their clients during the first wave of the COVID-19 pandemic. Of all professionals who have carried out tele-rehabilitation during the pandemic, 46% planned to use TR regularly or probably also after the pandemic. There were also clear differences between the professions. Psychotherapists carried out TR during the pandemic and planned to use it also after the pandemic more often than the other professional groups. The qualitative analysis revealed that therapists identified several beneficial but also multiple challenging features of TR. Psychotherapists reported less challenges than other professions. The pandemic has clearly sped up the use of TR in rehabilitation.

## 1. Introduction

On March 2020, the Finnish Government after approval by the Parliament of Finland issued a decree implementing the Emergency Powers Act due to the COVID-19 pandemic. Several measures were carried out until 13 May and even later. These included, for example, closing the schools, shutting down public facilities, reducing the number of participants in public meetings, obligating people over the age of 70 to avoid human contact if possible, closing the borders and setting quarantine rules [1]. These measures, in addition to the guidelines given by the Ministry of Social and Health Affairs, areal and local health care authorities as well as service provision organizations such as the Social Insurance Institution of Finland (Kela), which is the largest rehabilitation service organizer in Finland, naturally changed the implementation of rehabilitation services. To minimize contacts between clients and healthcare workers freelance rehabilitation professionals did not have access to social or health care institutions, residential homes for elderly and disabled people or schools for disabled people. Face-to-face rehabilitation services for people over 70 were, due to the limitations, nearly impossible. Overall, the situation limited the possibilities for in-person rehabilitation dramatically.

During the COVID-19 pandemic, tele-rehabilitation (TR) has been a widely recommended solution to reduce inpatient and outpatient visits and to continue the clients’ rehabilitation processes. The pandemic has forced professionals to use digital tools in their service provision. National professional unions, such as the Canadian Physiotherapy Association [2] and the Finnish Occupational Therapy Association [3] have provided examples and guidelines for tele-rehabilitation.

TR means provision of rehabilitation services at distance. TR refers to the use of different information and communication technologies (ICT), such as mobile phones, tablets, computers and TV applications, to provide rehabilitation services to people remotely in their homes or other environments [4,5]. There is plenty of evidence about the effectiveness of TR. For example, a systematic review on the efficacy of TR interventions among stroke survivors suggests that TR interventions have either better or equal salutary effects on motor, higher cortical, and mood disorders compared with conventional face-to-face therapy [6]. Another systematic review studied the effectiveness of physiotherapy with TR on postoperative functional outcomes and quality of life in surgical patients, and concluded that physiotherapy with TR has the potential to increase quality of life, is feasible, and is at least equally effective as usual care in surgical populations [7].

The Finnish national TR project 2016–2019 created recommendations for TR based on the results of the project [8] (pp. 289–293). The recommendations dealt with applicability, rationale, implementation, preconditions for initiation, support, technology and context of TR practice. For example, the applicability of TR for clients needs to be assessed individually. As a rationale, clients should have the possibility to choose TR. The implementation of TR should start with a face-to-face meeting, however, depending on the clients’ needs. Initiating TR requires proper preparation and training for all parties. Continuous IT support is necessary as well as new kinds of means to support clients. Before starting TR, it is necessary to ensure suitable functioning technology and information security. The TR context should provide information, education, and administrative support.

Despite positive research outcomes, the uptake of TR has been relatively slow until the COVID-19 pandemic. In Finland, even if TR has been under development in several projects since the year 2000, the regular use of TR has been quite low [9], although it increased towards the end of the 2010s. Results from a survey of rehabilitation clinicians in the United States show that 51% of the clinicians reported being comfortable integrating TR (mRehab) technology into their practice, and only 23% felt knowledgeable about available rehabilitation technology [10]. Barriers to used TR deal with doubts regarding the ease of using TR [11], problems in the uptake of TR [12], issues with technically challenged staff, resistance to change, cost, reimbursement and patient-related issues [13].

Research on TR during the COVID-19 pandemic most commonly describes the rehabilitation of COVID-19 patients [14], interventions for specific diagnoses [15], or the feasibility of and patient satisfaction with TR [16] or describes the experiences of clients with disabilities [17]. However, research comparing or describing viewpoints of different professional groups is scarce.

The aim of this study was to investigate and compare the uptake of TR in Finland amongst different rehabilitation professions during the COVID-19 pandemic. The professions included in the study were psychotherapists (PsT), physiotherapists (PT), occupational therapists (OT) and speech and language therapists (SLT). These are the largest rehabilitation professional groups that provide rehabilitation services in Finland. Research interests were to investigate quantitatively potential differences between professions in carrying out TR during the COVID-19 pandemic, in the plans to carry out TR after the pandemic, and whether these differences could be explained by work experience or familiarity with TR before the COVID-19 pandemic. In addition, the goal was to explore in more depth therapists’ views about the features that work and the challenges of TR by analyzing qualitative data.

## 2. Materials and Methods

### 2.1. Study Design and Data Collection Methods

This study is part of a larger study aiming to investigate the quality of individual therapies and change in the relationships between clients and therapists. Ethical approval for the main study (following the tenets of the World Medical Association Declaration of Helsinki) was obtained from the Ethical Review Board of the Social Insurance Institution of Finland in Helsinki. This study was approved as an additional element to the main study in the management group of Kela Research.

The survey collected quantitative data using closed-ended questions and qualitative data using open-ended questions. Respondents replied to the questionnaire anonymously, and a reply to the questionnaire was seen as informed consent from the participant. Data were collected from 7 May to 1 June 2020.

The questionnaire was composed of 24 questions about rehabilitation professionals’ work and use of TR during the COVID-19 pandemic. The questionnaire was specifically designed for the purposes of this study. The questionnaire was anonymized and did not contain any identifying information. Answering all questions was not mandatory. Background questions collected sociodemographic data (profession, age, sex, job experience and district), job situation and use of rehabilitation services during the COVID-19 pandemic.

Our outcome variables dealt with carrying out TR during COVID-19 and plans to use TR after COVID-19. The answers to the question “Have you carried out tele-rehabilitation during the corona pandemic?” were categorized as follows: “with most clients” and “with all clients” to 1 and “with some clients” and “not at all” to 0. The answers to the question “Based to your experiences now, do you plan to use tele-rehabilitation also after corona pandemic?” were classified “probably” and “yes, regularly” to 1 and “possibly” and “not at all” to 0. This question was posed only to those who carried out TR during the COVID-19 pandemic.

We used two background variables: work experience in years and familiarity with TR in the beginning of the COVID-19 pandemic (Table 1). Answers to the question “How familiar you were with tele-rehabilitation in the beginning of the corona pandemic?” were categorized as follows: (1) Entirely new practice, (2) I had the readiness for tele-rehabilitation, but no experience yet, (3) I had tried tele-rehabilitation with individual clients and (4) An established practice. The last two options were combined.

Two open-ended questions asked the therapists’ views about the features of TR that worked well and the features that were challenging in implementing TR.

### 2.2. Participants

Participants were recruited using e-mail addresses from the Kela directory. E-mail invitations were sent to 1687 therapists of whom 216 replied the survey via this data collection method. In addition, networks and newsgroups of professional organizations, and social media were used to recruit participants. This round of data collection produced 689 replies. Overall 905 replied the survey and 79% of them who were service providers for Kela. The online questionnaire was available to the respondents as a link on the Kela website. To meet the inclusion criteria, participants had to be licensed to practice psychotherapy, physiotherapy, occupational therapy, speech and language therapy, music therapy or rehabilitation neuropsychology, and currently practicing individual outpatient therapy services either in the public or private sector.

The survey garnered answers from 905 therapists, of whom 29% of them were psychotherapists, 28% physiotherapists, 22% speech and language therapists, 16% occupational therapists, 3% music therapists, and 2% rehabilitation neuropsychologists. In this study, we restricted the analysis to the four most common professions (*n* = 850).

The average age of the respondents was 49.7 years, and 93% were women. Over half of the respondents had over 20 years of work experience (Table 1). Physiotherapists were the most experienced, occupational therapists the least. For 58% of the respondents TR was an entirely new practice. Psychotherapists differed from other professions; half of them had tried or established practice before the COVID-19 pandemic.

### 2.3. Data Analysis

#### 2.3.1. Quantitative Analysis

Quantitative analyses were conducted using Stata 14.2. The differences in two outcomes between professions as well as outcomes by two background variables were studied by cross tabulation, and 95% confidence intervals were used to examine whether the differences were statistically significant.

A logistic regression model was used to study whether the differences between professions in two outcome variables could be explained by background variables. Model 1 included only professions. In model 2, work experience in years and familiarity with TR before the COVID-19 pandemic was controlled for. The same models were applied to both outcome variables. Physiotherapists were used as a reference group. The Akaike information criterion (AIC) and Bayesian information criterion (BIC) were used to estimate the quality of each model. A smaller AIC/BIC indicates a better-fitting model.

#### 2.3.2. Qualitative Analysis

For the qualitative part of the study, hermeneutics was selected as an appropriate research approach since the research goal was to gain deeper understanding perspectives of well-working and challenging features of TR [18]. The qualitative data-analysis of the study sought the views of four different therapy professions about TR. The responses to two open ended questions (well-working and challenging features of TR) were split up according to the professions. The question related to the well-working features of TR received 626 responses, of which 131 were from PTs, 99 from OTs, 175 from SLTs and 223 from PsTs. The question dealing with the challenges of TR received 676 responses, of which 145 were from PTs, 114 from Ots, 181 from SLTs and 236 from PsTs. Overall, the data were rich, including detailed descriptions and perceptions.

Deductive content analysis was employed in this study [19,20]. The recommendations for TR from the Finnish national TR project 2016–2019 [8], were used as the coding schema in the data analysis. The coding schema included seven themes: (1) participant group, (2) rationale for TR, (3) implementation of TR, (4) preconditions for the initiation of TR, (5) support during TR, (6) technology and the (7) context of TR practice. All themes of the coding scheme were relevant for the data analysis of this study. Data analysis also produced four additional themes providing insight into the therapists’ perspectives on TR practice.

Two researchers (SM and TH) conducted the qualitative analysis. In the first phase, both researchers independently open coded responses given by two professions. Secondly, these researchers independently classified the codes related to features working well in TR and the challenges of TR according to the coding schema. In the third phase, they generated sub-themes from the themes together. After classifying the data according to sub-themes, both researchers reviewed all sub-themes. To enhance trustworthiness and the credibility of the analysis, the discrepancies were discussed and agreed upon. Researchers discussed frequently to further consider their interpretations of the coded text. Finally, the whole research group checked the sub-themes.

## 3. Results

### 3.1. Uptake of Tele-Rehabilitation amongst Rehabilitation Professionals

Over half of all respondents carried out TR with all or most of their clients during the COVID-19 pandemic. However, there were large differences between professions (Table 2). Most PsTs carried out TR with most or all of their clients. The proportion was lowest amongst the PTs.

Based on their experiences during the COVID-19 pandemic, almost half of the professionals among those who carried out TR during the pandemic planned to use TR regularly or probably also after the pandemic. Again, there were differences between professions (Table 2). Psychotherapists planned to use TR more often than other professions. Half of the speech and language therapists and a third of the occupational therapists and physiotherapists planned to use TR regularly or probably.

The proportion of those professionals who carried out TR with most or all clients was lowest among those with over 30 years of work experience compared to those with fewer years (Table 2). Those who were familiar with TR before the COVID-19 pandemic carried out TR during the pandemic more often than those without earlier experience.

Therapists with the most work experience reported to be the most unlikely group to use TR after the COVID-19 pandemic. The more familiar TR was before the COVID-19 pandemic, the more common was its planned use after the pandemic.

Whether the differences between professions in carrying out TR during the COVID-19 pandemic could be explained by background variables was studied with a logistic regression model (Table 3). In model 1, the odds ratio (OR) for occupational therapists was 3.1 and for speech and language therapists 11.8 compared to physiotherapists (the reference group). Psychotherapists had an almost 30 times higher OR to carry out TR during the COVID-19 pandemic in comparison to physiotherapists. Controlling for work experience in years and familiarity with TR before the COVID-19 pandemic did not notably reduce the differences (model 2).

The odds ratio for planning to use TR after the COVID-19 pandemic was highest among psychotherapists, whose OR was 2.6 compared to the reference group of physiotherapists (Table 4). Speech and language therapists also had a statistically significantly higher odds ratio, whereas occupational therapists did not differ from the reference group. Adjusting for work experience and familiarity with TR before the COVID-19 pandemic explained part of the differences

### 3.2. Working Features and Challenges Related to TR

The qualitative data analysis produced four new themes in addition to the themes within the coding schema, producing 11 themes overall (see Table 5). Within three of the themes, representatives of all professions had similar views. Within eight themes, the views varied between professions. However, the experiences of representatives within specific professional groups were not identical and there was plenty of individual variation. The experience of using TR is individual. In addition, the analysis revealed that the same features related to TR can been experienced as both working and challenging. The features that worked well with TR were experienced more uniformly than its challenges.

Firstly, we present the findings related to the themes where professions had similar views. After that, we will report the findings from the themes on which the views varied between professions in more detail. The number of well-working and challenging sub-themes by professional groups is presented in Table 6 (sub-themes are presented in more detailed in Table S1).

The majority of therapists perceived ***the applicability of and the rationale for TR*** in a similar way and there were no distinct differences in the views of representatives of different professions, such as in age or diagnostic group of clients. No disease groups were excluded from TR. The common opinion was that the suitability of TR needs to be assessed individually, and TR is not suitable when starting therapy with a new client. The findings show that TR may not be appropriate for clients with complex diseases and health situations that require a great variety of health services. As the rationale for TR, in the context of this study, the main reason for tele-rehabilitation was COVID-19 and the clients’ need for rehabilitation.

The theme of ***engagement with and motivation for tele-rehabilitation*** emerged and was experienced by representatives of all professions in a similar way. The findings reveal that clients and their close associates were mainly committed to TR. New methods increased motivation. However, the lack of motivation was also noted and, in some cases, the difficulty to maintain motivation during therapy sessions.

”Motivating course of action, new task types, temporally enables more frequent work with the client, parents more committed to therapy.” Occupational therapist.

***The implementation of TR*** included 16 sub-themes (see Table 6). Based on the experiences of the therapists, five sub-themes were identified to work well, of which four were shared between representatives of all professions. These were the negotiations and discussions with clients, use of familiar methods and materials, use of guidance and clients’ progress in therapy. Negotiations and discussions were considered both as features that work well, as they strengthen collaboration with clients, and as a challenge because rehabilitation did not take place in daycare or school settings and some OTs and SLTs experienced negotiations in TR superficial.

Overall, there was more variation amongst professions in challenges related to the implementation of TR. One sub-theme that dealt with physical exercises and manual and concrete guidance of clients and their close associates was identified as challenging by all the professions. PTs, OTs and SLTs considered assessment and observations of a client’s activity, as well as limited methods and tasks as challenging. However, representatives of the same professions also considered diversity and new kinds of methods and perspectives as well-working features of TR. OTs identified activity modification during therapy sessions and animal assisted therapy as demanding. SLTs reported the largest number of challenges, amongst them auditive and visual accuracy during observations, tactile feeding rehabilitation and adherence to goals. Shared challenges between SLTs and PsTs were use and distribution of materials and physical exercises and activities. Both OTs and PsTs considered working with emotions challenging in TR.

*“If activities planned for therapy are not enough, for example, tasks are completed faster than usual or not succeeding at all, coming up with extra doing has been challenging for me.” Occupational therapist.*

***Initiating TR*** included 13 sub-themes (Table 6). Of the six sub-themes related to working features when initiating TR, only one—clients’ commitment, positive attitude and motivation towards TR—was shared between representatives of all professions. In addition PsTs named as well-working features shared challenges in the uptake of TR, collectively agreed schedules and procedures and therapists’ positive attitude. Two sub-themes out of eight relating to challenges in initiating TR were common for representatives of all professions. They were learning new ways to work and prejudices towards TR. OTs, SLTs and PsTs considered additional procurements for TR challenging, whereas OTs and PTs identified that lack of skills and knowledge challenge initiation of TR. PTs and SLTs named motivating clients to try TR as a challenge. SLTs considered that licensure and legal liability as well as confusing administrative instructions make TR demanding.

*“In the beginning, time and money went into learning and taking over platforms. This also creates additional costs.” Physiotherapist.*

Within the theme of ***support during TR*** none of the nine sub-themes (Table 6) were shared between all professions. PTs, OTs and SLTs identified support provided for the professionals and clients’ skilled personal assistance as features that support well-working TR. However, these features as well as personal assistance for the client were considered both as well-working and as challenging feature depending on their availability. Lack of support was identified as a challenge by PTs and OTs, and lack of interpretation services, by OTs and SLTs. OTs, SLTs and PsTs identified challenging the therapist’s role as technical support for client.

*“The client has had no access to an assistant and movements cannot be executed independently or the assistant has not had sufficient competence in the situation.” Physiotherapist.*

The representatives of all professions experienced ***technology*** quite consistently (see Table 6). Seven sub-themes were identified within the theme of technology. The sub-theme of technology and bandwidth was shared between all professions, both as well-working and challenging, depending on its’ functioning and reliability. In a similar way, availability or lack of appropriate and easy to use equipment and applications supports or challenges TR. PTs, OTs and SLTs identified that poor video and audio quality as well as poor device positioning and lightning conditions challenge TR. Information security was considered as a challenge by OTs and PsTs.

*“Not everyone has suitable device at home other than a smartphone. On that it’s hard to see shared tasks or otherwise establish good contact.” Speech and language therapist.*

***The context of TR practice*** includes nine sub-themes (Table 6). One of the four sub-themes that work in a TR context, flexibility in planning and carrying out TR, was shared between the professions. However, PTs, OTs and PsTs experienced flexible schedules also as a challenge, as longer workdays and changing schedules caused burden and mixing home and work affairs. In a same way PTs, OTs and SLTs identified that TR practice supports efficient use of resources and on the other hand planning and preparing sessions was considered as a time consuming challenge. None of the five sub-themes of challenges related to TR were shared between professions.

*“No cancellations due to illness or fatigue.” Speech and language therapist.*

The following three themes are additional to the pre-defined coding schema and emerged during data analysis.

***The professionals’ well-being at work during TR*** includes seven sub-themes (Table 6). Only SLTs and PsTs expressed sub-themes that work in TR, which were their development in ways guiding clients, learning new and decreased emotional strain. Representatives of all professions shared one of the four sub-themes that deal with challenges. They felt that TR burdens and exhausts more than face-to-face rehabilitation. In addition, OTs and SLTs pointed out experiences of loneliness at work. Concern about clients for OTs and additional challenges at work for PTs were also challenging.

*“It’s been actually interesting and challenging, it’s been great to find out it works like this as well”. Physiotherapist.*

***Interaction during TR*** raised plenty of views (see Table 6). Two of the six sub-themes that work in interaction during TR, fluent and intensive interaction and clients’ active engagement, were shared between representatives of all professions. Two sub-themes, clients’ better possibility of controlling interaction and the opportunity to focus on essential issues in therapy sessions, were identified only by PsTs.

From 11 of the sub-themes of challenges related to interaction during TR, representatives of all professions shared three: differences and intensity of interaction, no shared environment and a therapeutic relationship. Two sub-themes, work with emotions and clients’ preference for personal presence, were expressed only by PsTs. PTs, OTs and SLTs identified misunderstandings in communication, difficulties in guiding interaction between the client and close associate and lack of physical contact as challenging. OTs, SLTs and PsTs again named understanding nonverbal and visual cues as challenging.

*“In telerehabilitation one is more tied to what’s spoken; observing the mood of the meeting and the gestures and state of the client is a little more difficult.” Psychotherapists.*

***TR in the everyday life environment*** includes 15 sub-themes (Table 6). Of the five sub-themes that work, two were shared between representatives of all professions. The analysis brought up that during TR therapists can learn more about their clients in their everyday life environment, and they are able to apply therapy within everyday life. In addition, they consider it easier for clients to participate in therapy. Furthermore, PTs, OTs and SLTs considered that everyday life environment is good for participation of close associates, and PTs, OTs and PsTs noted that it is safe for the clients.

*“It’s been amazing to get into the clients’ own environment” Psychotherapists.*

The number of sub-themes related to challenges was much greater. Encountered challenges were connected to the physical environment (for example, lack of space or therapeutic equipment, no quiet and calm place for meetings). PTs and OTs noted worries about the safety of the home environment. Part of the experienced challenges at home were connected to other persons, such as lack of peaceful space and lack of privacy. Different profession groups expressed varying views about the everyday life context. Psychotherapists expressed only three challenges. Participation of close associates was identified as challenging by PTs, PTs and SLTs as it does change interaction.

## 4. Discussion

The results of this study show that over half of the physiotherapists, occupational therapists, speech and language therapists as well as psychotherapists who participated in this study did take up TR with all or most of their clients during the first wave of the COVID-19 pandemic. The pandemic clearly sped up the use of TR in rehabilitation. Most therapists planned to use TR also after the COVID-19 pandemic, when face-to-face therapies are again safe to carry out. However, there were clear differences between the professions. Psychotherapists carried out TR more and also planned to use TR in the future more than the other professional groups. The therapists with the longest work experience reported to be more unlikely to use TR after the COVID-19 pandemic than others. The therapists identified multiple well-working and challenging features of TR. Overall, some views regarding the features were common for representatives of all the professions, but the differences were greater. Again, psychotherapists reported less challenges than the other professions.

Research comparing or describing the viewpoints of different professional groups in the use of TR is scarce. Dahl-Popolizio et al. [21] explored how occupational therapists experience using TR during COVID-19. The aim of the study by Kraljević et al. [22] was to gain an insight into how speech and language pathologists in Croatia coped with the implementation of telepractice during COVID-19 [22]. Tenforde et al. [16] described physical, occupational, and speech therapy patients’ satisfaction towards TR.

Psychotherapy, when compared to other therapies, seems to be easier to conduct digitally, e.g., by use of mobile phone only, although psychotherapists considered working with emotion challenging. This may also be due to differences in the recipients of TR, such as age and education. PsT clients have less physical disabilities compared to the clients of other therapies, therefore the therapy sessions are easier to conduct and there is usually no need for assistance. However, PT, OT and SLT clients may need a lot of assistance in TR. Especially TR with children requiring help from the parents or other adults. Speech and language therapists and occupational therapists pointed out that in TR sessions the parents were involved in the therapeutic relationship, unlike in face-to-face therapy, where parents are not so intensively involved. These results are in line with studies by Cacioppo et al. [17] and Dahl-Popolizio et al. [21] that emphasize that parents or caregivers needed to engage in the TR sessions to ensure client participation. Physical, occupational, and speech and language therapy differ from psychotherapy in methods, used tools and activities, and therefore technological challenges in TR are greater for the former professions. These technical challenges, identified also by Tenforde et al. [16], include difficulties with camera/device positioning and video/audio quality. Furthermore, TR is not necessarily feasible or suitable for people with complex diseases and life situations [23]. Additionally, Kraljević et al. [22] pointed out that certain clients could not be included in TR due to the client’s dependence on other family members due to a lack of digital competence.

For physiotherapists, who typically base the therapy on physical contact and conduct part of physical therapy manually, TR was challenging and they conducted TR less compared to other professions in this study.

The therapists who participated in this study exceeded the barriers related to TR by making the clients’ needs their priority during the pandemic. Still, they reported a number of challenges, many of which are similar to the barriers of TR identified in the systematic review by Kruse et al. [12]. These were, e.g., legal liability, privacy and confidentiality concerns, time consumption and resistance to change.

Some features of TR were considered to both well-working and challenging in TR. For example, possibilities for more flexible daily schedules were considered positive, but also caused burden, because workdays became longer and home and work affairs were mixed. Furthermore, all professional groups considered TR to be more exhausting than face-to-face therapy, and feelings of loneliness at work affected therapists’ well-being at work.

Learning new skills may cause burden that may be reduced with experience. Kraljević et al. [22] found out that after a short period of work under new conditions, speech and language therapists adjusted and started gaining competence in TR. At the same time, their satisfaction with TR increased. Results also indicate that a lack of competence relates with lesser satisfaction with TR. Competence with telepractice requires additional training [22]. Therapists who were familiar with TR before the COVID-19 pandemic carried out TR during the pandemic more often than those with no earlier experience.

The results of this study on the uptake of TR during COVID-19 are in line with recent studies from the United States and Croatia. In Croatia, 71% of speech and language therapists offered TR to all their clients during the ongoing COVID-19 pandemic [22]. In the United States, the use of telepsychology increased 12-fold during the pandemic, and 26-fold in outpatient treatment facilities [24]. Although the professional groups from our study are not fully comparable, 67% of psychologists in the US conducted their clinical work with telepsychology during the pandemic, whereas 84% of the psychotherapists who participated in our study carried out TR with most or all of their clients. Similarly, psychologists in the US projected that they would perform 35% of their clinical work via telepsychology after the pandemic, while 56% from the psychotherapists in our study planned to use TR regularly or probably.

Overall, the data of the study was numerous, and the qualitative data was rich and manifold. The quantitative and qualitative data complement each other. The qualitative data helped to gain a deeper understanding of therapist-perceived features in delivering TR. The questionnaire was available online and therefore the representativeness of the respondents could not be ensured. The responses may have been biased against or in favor of TR. However, the large number of responses may attenuate the bias. More detailed characteristics of respondents would have been useful. For example, more detailed information of the respondents’ therapeutic practices could have provided more insight to the applicability of TR.

The recommendations for TR [8] that were used as the coding schema in this study, added with additional themes which emerged, may provide a good concept for planning and implementing TR. These recommendations point out critical issues of practicing TR. By using the coding schema it was possible to identify and describe both well-working and challenging features of TR.

The professional groups who participated in the study can use the results to develop and improve TR practices within the profession. Furthermore, the results may help rehabilitation professionals to identify challenges that limit clients’ participation in TR. The positive outcome about the uptake of TR encourages including TR in all therapies as a part of the intervention process. However, starting a new therapy process with a new client with TR is not recommended. The results of this study support the continued use of tele-rehabilitation by OTs, PTs, SPTs and PsTs as a suitable service delivery model. These findings are consistent with a recent study of OTs’ perspectives of TR [19]. A rehabilitation model where both face-to-face and telepractice sessions will be used may become the norm in the future [8,25].

This study adds to a limited body of knowledge of TR adoption and challenges to the same by comparing professionals’ views of TR in the first phase of the COVID-19 pandemic. In the future, research is needed to examine clients’ experiences of TR, as well as perspectives of families and close associates.

## 5. Conclusions

The COVID-19 pandemic sped up the use of TR in rehabilitation. Therapists regarded TR positively and were able to take up TR rapidly during the societal crisis caused by COVID-19. However, for the proper uptake of TR, therapists need both technical and practical support, training and time to adopt new technologies and design new materials required in TR. These may reduce the burden that therapists described when comparing TR during COVID-19 to their earlier experiences with face-to-face rehabilitation. The profession-related requirements in the use of TR need to be taken into account when planning TR.

## Figures and Tables

**Table 1:** Background variables (%) by profession.

	Physiotherapists	Speech and Language Therapists	Occupational Therapists	Psychotherapists	All Therapists
**Work experience in years**					
1–9	17	25	28	19	21
10–19	20	30	34	25	26
20–29	27	33	24	28	28
30+	37	12	14	28	24
Total					
%	100	100	100	100	100
N	246	197	139	259	841
**Familiar with TR before COVID-19**					
Entirely new practice	65	75	64	34	58
Readiness but no experience	25	14	28	17	20
Had tried/established practice	10	11	8	49	22
Total					
%	100	100	100	100	100
N	247	198	140	262	847

**Table 2:** Proportions of those who carried out tele-rehabilitation with most/all clients during the COVID-19 pandemic among all therapists and proportions of those who probably or regularly plan to use tele-rehabilitation also after the COVID-19 pandemic among those who carried out tele-rehabilitation, number, percentage (%) and 95% confidence intervals (CI).

Background	Carried Out TR with Most/All Clients during the COVID-19 Pandemic, among All Therapists		Planning to Use TR Probably/Regularly after the COVID-19 Pandemic, among Those Who Carried Out TR	
	% (N)	95% CI	% (N)	95% CI
**Profession**				
Physiotherapists	15 (250)	(10–19)	34 (160)	(27–42)
Speech and language therapists	68 (198)	(61–74)	48 (184)	(41–56)
Occupational therapists	36 (140)	(28–44)	37 (121)	(29–46)
Psychotherapists	84 (262)	(80–88)	56 (257)	(50–62)
**Work experience in years**				
1–9	58 (177)	(50–65)	51 (156)	(43–59)
10–19	58 (222)	(52–65)	53 (197)	(46–60)
20–29	50 (238)	(44–57)	47 (207)	(40–54)
30+	43 (204)	(36–49)	32 (155)	(25–40)
**Familiar with TR before COVID-19**				
Entirely new practice	45 (488)	(41–50)	34 (402)	(29–39)
Readiness but no experience	47 (173)	(39–54)	49 (147)	(41–57)
Had tried/established practice	75 (186)	(68–81)	72 (172)	(65–78)
Total	52 (850)	(49–55)	46 (722)	(42–50)

**Table 3:** Carried out tele-rehabilitation with most/all clients during the COVID-19 pandemic by profession, odds ratios (OR) and 95% confidence intervals (CI) (*n* = 837).

Profession	Model 1		Model 2	
	OR	95% CI	OR	95% CI
Physiotherapists	1.00		1.00	
Speech and language therapists	11.84	(7.47–18.77)	11.23	(6.99–18.03)
Occupational therapists	3.13	(1.91–5.12)	2.80	(1.69–4.63)
Psychotherapists	28.77	(17.78–46.55)	24.80	(14.97–41.09)
Akaike information criterion (AIC)	873		871	
Bayesian information criterion (BIC)	1021		914	

Model 1: Occupation, Model 2: Model 1 + work experience in years + familiar with TR before COVID-19 pandemic.

**Table 4:** Planning to use tele-rehabilitation also after COVID-19 pandemic probably/yes, regularly by profession among those who carried out tele-rehabilitation during the COVID-19 pandemic, odds ratios (OR) and 95% confidence intervals (CI) (*n* = 741).

Profession	Model 1		Model 2	
	OR	95% CI	OR	95% CI
Physiotherapists	1.00		1.00	
Speech and language therapists	1.88	(1.23–2.89)	1.79	(1.13–2.82)
Occupational therapists	1.23	(0.76–1.99)	1.05	(0.63–1.74)
Psychotherapists	2.55	(1.71–3.81)	1.46	(0.93–2.29)
Akaike information criterion (AIC)	1003		928	
Bayesian information criterion (BIC)	1021		969	

Model 1: Occupation, Model 2: Model 1 + work experience in years + familiar with TR before COVID-19 pandemic.

**Table 5:** The themes of the qualitative analysis.

Themes of the Coding Schema *	
Applicability of TR	All professions had similar views
Rationale for TR	
Implementation of TR	Professions had differing views
Preconditions for initiating TR	
Support during tele-rehabilitation	
Technology	
Context of TR practice	
**Additional Emerged Themes**	
Engagement and motivation to TR	All professions had similar views
The professionals’ well-being at work during TR	Professions had differing views
Interaction during TR	
TR in the everyday life environment	

* Adapted from Salminen and Hiekkala ([8], pp. 289–293).

**Table 6:** The number of well-working and challenging sub-themes by professional group.

**Implementing Tele-Rehabilitation**											
Well-working	PT	OT	SLT	PsT	All sub-themes	Challenging	PT	OT	SLT	PsT	All sub-themes
	5	5	5	4	5		4	7	9	3	11
**Initiating Tele-Rehabilitation**											
Well-working	PT	OT	SLT	PsT	All sub-themes	Challenging	PT	OT	SLT	PsT	All sub-themes
	1	4	3	4	6		5	5	6	3	7
**Support during Tele-Rehabilitation**											
Well-working	PT	OT	SLT	PsT	All sub-themes	Challenging	PT	OT	SLT	PsT	All sub-themes
	2	2	3	0	3		3	5	4	2	6
**Tele-Rehabilitation Technology**											
Well-working	PT	OT	SLT	PsT	All sub-themes	Challenging	PT	OT	SLT	PsT	All sub-themes
	2	2	2	2	2		4	4	4	2	5
**The Context of Tele-Rehabilitation Practice**											
Well-working	PT	OT	SLT	PsT	All sub-themes	Challenging	PT	OT	SLT	PsT	All sub-themes
	3	2	3	3	4		2	3	3	2	5
**Professionals’ well-being at Work**											
Well-working	PT	OT	SLT	PsT	All sub-themes	Challenging	PT	OT	SLT	PsT	All sub-themes
	0	0	2	1	3		2	3	2	1	4
**Interaction during Tele-Rehabilitation**											
Well-working	PT	OT	SLT	PsT	All sub-themes	Challenging	PT	OT	SLT	PsT	All sub-themes
	3	4	4	5	6		6	8	9	6	11
**Tele-Rehabilitation in Everyday life Environment**											
Well-working	PT	OT	SLT	PsT	All sub-themes	Challenging	PT	OT	SLT	PsT	All sub-themes
	5	5	3	3	5		8	7	6	3	10
Number of well-working sub-themes all together	PT	OT	SLT	PsT	All sub-themes	Number of challenging sub-themes all together	PT	OT	SLT	PsT	All sub-themes
	21	24	25	22	34		34	42	43	22	59

PT = Physiotherapists, OT = Occupational therapists, SLT = Speech and language therapists, PsT = Psychotherapists.

## Data Availability

Not available due to ethical reasons.

## Supplementary Materials

The following are available online at https://www.mdpi.com/article/10.3390/ijerph18084383/s1, Table S1: Well-working and challenging sub-themes of tele-rehabilitation, by professional group.

## References

[1] Information and Advice on the Coronavirus. Government Press Releases. https://valtioneuvosto.fi/en/information-on-coronavirus/press-conferences.

[2] (2021). Tele-Rehabilitation. https://physiotherapy.ca/tele-rehabilitation.

[3] Etätoimintaterapia Suomen Toimintaterapeuttiliitto ry. http://www.toimintaterapeuttiliitto.fi/etatoimintaterapia/.

[4] Brennan D.M., Mawson S., Brownsell S. (2009). Telerehabilitation: Enabling the remote delivery of healthcare, rehabilitation and self management. Stud. Health Technol Iinform.

[5] Salminen A.-L., Heiskanen T., Hiekkala S., Naamanka J., Stenberg J.-H., Vuononvirta T., Salminen A.-L., Hiekkala S., Stenberg J.-H. (2016). Etäkuntoutuksen ja siihen läheisesti liittyvien termien määrittelyä. Etäkuntoutus.

[6] Safro F., Ulasavets U., Opare-Sem O., Ovbiagele B. (2018). Tele-rehabilitation after stroke. An updated systematic review of the literature. J. Stroke Cerebrovasc. Dis..

[7] van Egmond M.A., van der Schaaf M., Vredeveld T., Vollenbroek-Hutten M.M.R., van Berge Henegouwen M.I., Klinkenbijl J.H.G., Engelbert R.H.H. (2018). Effectiveness of physiotherapy with telerehabilitation in surgical patients: A systematic review and meta-analysis. Physiotherapy.

[8] Salminen A.-L., Hiekkala S., Salminen A.-L., Hiekkala S. (2019). Suosituksen etäkuntoutukseen. Kokemuksia Etäkuntoutuksesta. Kelan Etäkuntoutushankkeen Tuloksia.

[9] Vuononvirta T., Salminen A.-L., Hiekkala S., Stenberg J.-H. (2016). Etäkuntoutus Suomessa. Etäkuntoutus.

[10] Morris J., Jones M., Thompson N., Wallace T., DeRuyter F. (2019). Clinician Perspectives on mRehab Interventions and Technologies for People with Disabilities in the United States: A National Survey. Int. J. Environ. Res. Public Health.

[11] Pierce B.S., Perrin P.B., McDonald S.D. (2020). Path analytic modeling of psychologists’ openness to performing clinical work with telepsychology: A national study. J. Clin. Psychol..

[12] Brouns B., Meesters J.J.L., Wentink M.M., de Kloet A.J., Arwert H.J., Vliet Vlieland T.P.M., Boyce L.W., van Bodegom-Vos L. (2018). Why the uptake of eRehabilitation programs in stroke care is so difficult-a focus group study in the Netherlands. Implement. Sci..

[13] Kruse C.S., Karem P., Shifflett K., Vegi L., Ravi K., Brooks M. (2018). Evaluating barriers to adopting telemedicine worldwide: A systematic review. J. Telemed. Telecare.

[14] Rosen K., Patel M., Lawrence C., Mooney B. (2020). Delivering Telerehabilitation to COVID-19 Inpatients: A Retrospective Chart Review Suggests It Is a Viable Option. HSS J..

[15] Molinaro A., Micheletti S., Pagani F., Garofalo G., Galli J., Rossi A., Fazzi E., Buccino G. (2020). Action Observation Treatment in a tele-rehabilitation setting: A pilot study in children with cerebral palsy. Disabil. Rehabil..

[16] Tenforde A.S., Borgstrom H., Polich G., Steere H., Davis I.S., Cotton K., O’Donnell M., Silver J.K. (2020). Outpatient Physical, Occupational, and Speech Therapy Synchronous Telemedicine: A Survey Study of Patient Satisfaction with Virtual Visits during the COVID-19 Pandemic. Am. J. Phys. Med. Rehabil..

[17] Cacioppo M., Bouvier S., Bailly R., Houx L., Lempereur M., Mensah-Gourmel J., Kandalaft C., Varengue R., Chatelin A., Vagnoni J. (2020). Emerging health challenges for children with physical disabilities and their parents during the COVID-19 pandemic: The ECHO French survey. Ann. Phys. Rehabil. Med..

[18] Tan H., Wilson A., Olver I. (2009). Ricoeur’s Theory of Interpretation: An Instrument for Data Interpretation in Hermeneutic Phenomenology. Int. J. Qual. Methods..

[19] Elo S., Kyngäs H. (2008). The qualitative content analysis process. J. Adv. Nurs..

[20] Mojtaba V.M., Turunen H., Bondas T. (2013). Content analysis and thematic analysis: Implications for conducting a qualitative descriptive study. Nurs. Health Sci..

[21] Dahl-Popolizio S., Carpenter H., Coronado M., Popolizio N.J., Swanson C. (2020). Telehealth for the Provision of Occupational Therapy: Reflections on Experiences During the COVID-19 Pandemic. Int. J. Telerehabilit..

[22] Kraljević J.K., Matić A., Dokoza K.P. (2020). Telepractice as a reaction to the covid-19 crisis: Insights from Croatian SLP settings. Int. J. Telerehabilit..

[23] Leocani L., Diserens K., Moccia M., Caltagirone C. (2020). Disability through COVID-19 pandemic: Neurorehabilitation cannot wait. Eur. J. Neurol..

[24] Pierce B.S., Perrin P.B., Tyler C.M., McKee G.B., Watson J.D. (2020). The COVID-19 Telepsychology Revolution: A National Study of Pandemic-Based Changes in U.S. Mental Health Care Delivery. Am. Psychol..

[25] Camden C., Silva M. (2021). Pediatric Teleheath: Opportunities Created by the COVID-19 and Suggestions to Sustain Its Use to Support Families of Children with Disabilities. Phys. Occup. Ther. Pediatrics.

