# Supplementary material for: Uptake of Tele-Rehabilitation in Finland amongst Rehabilitation Professionals during the COVID-19 Pandemic
Source: Int J Environ Res Public Health. 2021 Apr 20;18(8):4383. doi: 10.3390/ijerph18084383 (PMC8074751; doi:10.3390/ijerph18084383)
Supplement: Supplementary file 1 [file ijerph-18-04383-s001.zip › ijerph-1124442-supplementary.pdf]

Table S1. Well-working and challenging sub-themes of tele-rehabilitation, by professional group.

**Implementing tele-rehabilitation**									
**Well-working**	**PT**	**OT**	**SLT**	**PsT**	**Challenging**	**PT**	**OT**	**SLT**	**PsT**
Negotiations, discussions and strengthened collaboration with clients	x	x	x	x	Assessment and observation of client's activity	x	x	x	
Use of familiar methods and materials	x	x	x	x	Physical exercises and activities, manual and concrete guidance of clients and their close associates	x	x	x	x
Use of guidance	x	x	x	x	Work with emotions		x		x
Clients' progress in therapy	x	x	x	x	Use and distribution of materials			x	x
New kinds of models, methods, techniques and perspectives	x	x	x		Negotiations and co-operation with home, daycare and school		x	x	
					Limited methods and techniques, focus on tasks and exercises	x	x	x	
					Activity modification and spontaneity in selection of exercises during therapy sessions		x	x	
					Animal assisted therapy		x		
					Auditive and visual accuracy during observations	x		x	
					Tactile feeding rehabilitation			x	
					Adherence to goals			x	
**Initiating tele-rehabilitation**									
**Well-working**	**PT**	**OT**	**SLT**	**PsT**	**Challenging**	**PT**	**OT**	**SLT**	**PsT**
Clients' commitment, positive attitude and motivation towards TR	x	x	x	x	Learning new ways to work	x	x	x	x
Shared challenges in the uptake of TR			x	x	Prejudices and attitudes of both professionals and clients	x	x	x	x
Successful rapid implementation		x			Rapid, unpredicted implementation		x	x	x
Starting with collectively agreed schedules and procedures		x		x	Additional procurements	x	x	x	
Available education and training		x	x		Lack of skills and knowledge	x	x		
Therapists' positive attitude				x	Motivating clients to try TR	x		x	
					Licensure, legal liability and confusing administrative instructions			x	
**Support during tele-rehabilitation**									
**Well-working**	**PT**	**OT**	**SLT**	**PsT**	**Challenging**	**PT**	OT	SLT	PsT

Support provided for the professionals	x	x	x		Lack of support for clients	x	x		
Skilled personal assistance for the client	x	x	x		Lack of skilled personal assistance for the client	x	x	x	
Parents' decent technical skills			x		Lack of interpretation services		x	x	
					Clients' lack of financial resources for necessary equipment			x	
					Therapist's role as a technical support for clients		x	x	x
					Lack of technical and administrative support for professionals	x	x		x
**Tele-rehabilitation technology**									
**Well-working**	**PT**	**OT**	**SLT**	**PsT**	**Challenging**	**PT**	**OT**	**SLT**	**PsT**
Functioning technology and bandwidth	x	x	x	x	Unreliable bandwidth and technology	x	x	x	x
Appropriate, suitable and easy to use applications and equipment	x	x	x	x	Information security		x		x
					Lack of appropriate devices	x	x	x	
					Poor video and audio quality.	x	x	x	
					Poor device positioning and lighting conditions	x		x	
**The context of tele-rehabilitation practice**									
**Well-working**	**PT**	**OT**	**SLT**	**PsT**	**Challenging**	**PT**	**OT**	**SLT**	**PsT**
Flexibility in planning and carrying out TR	x	x	x	x	Burden caused by flexible schedules, extended working days and new/additional duties at work	x	x		x
Efficient use of resources	x	x	x		Planning and preparing sessions and the production of materials is time consuming	x	x	x	
Supports regularity in therapy (Less client delays and cancellations)	x			x	Financial compensation is not in balance with the workload			x	
Increase in possibilities to implement therapy			x	x	Work and home affairs get mixed				x
					The use of certain assessment methods or standardized tests or diagnostic tools is not applicable in TR			x	
**Tele-rehabilitation related to the professionals' well-being at work**									
**Well-working**	**PT**	**OT**	**SLT**	**PsT**	**Challenging**	**PT**	**OT**	**SLT**	**PsT**
Development in ways guiding clients			x		TR burdens and exhausts more than face to face rehabilitation	x	x	x	x

Getting new knowledge, learning new things and ideas is refreshing			x		Experience of loneliness at work		x	x	
Decreased emotional strain				x	Concern about clients		x		
					Additional challenges at work	x			
**Interaction during tele-rehabilitation**									
**Well-working**	**PT**	**OT**	**SLT**	**PsT**	**Challenging**	**PT**	**OT**	**SLT**	**PsT**
Fluent and intensive interaction	x	x	x	x	Differences in interaction and its intensity compared to face to face situation	x	x	x	x
Clients' active engagement and improved focusing	x	x	x	x	Misunderstandings in communication	x	x	x	
Progress in interaction	x	x	x		Difficulties to guide interaction between client and close associate	x	x	x	
Encouraged clients		x	x	x	Time-consuming to learn to interact within an unfamiliar setting.		x	x	
Clients have better possibilities to control interaction.				x	Understanding nonverbal and visual cues		x	x	x
Possibility to focus on essential issues in therapy sessions				x	No shared physical environment	x	x	x	x
					Therapists' help, therapeutic alliance and confidentiality	x	x	x	x
					Work with emotions				x
					Clients' preference for personal presence				x
					Client too timid or passive to participate			x	
					Lack of physical contact	x	x	x	
**Tele-rehabilitation in everyday life environment**									
**Well-working**	**PT**	**OT**	**SLT**	**PsT**	**Challenging**	**PT**	**OT**	**SLT**	**PsT**
Therapists learn more about clients' everyday life environment and are able to apply methods in it	x	x	x	x	Lack of a peaceful space	x	x	x	x
Easy for clients to participate in the therapy sessions	x	x	x	x	Lack of capacity or resources in families to participate in TR	x	x	x	
Safe environment for the clients	x	x		x	Parental burden	x	x	x	
Participation of close associates	x	x	x		Lack of privacy and security		x		x
Clients take more responsibility for their own rehabilitation	x	x			Too little space and lack of safe environment for practical exercises at home	x	x		
					Lack of equipment or challenges in their use	x	x		
					Distribution of work amongst people present in therapy sessions	x		x	

	Participation of close associates changes interaction	x	x	x	
	Parents’ attitudes	x		x	
	Transfers to therapists’ reception that support orientation to therapy are missing				x

PT= Physiotherapists, OT= Occupational therapists, SLT= Speech and language therapists, PsT= Psychotherapists
